# Model-Augmented Haptic Telemanipulation: Concept, Retrospective Overview, and Current Use Cases

**DOI:** 10.3389/frobt.2021.611251

**Published:** 2021-06-11

**Authors:** Thomas Hulin, Michael Panzirsch, Harsimran Singh, Andre Coelho, Ribin Balachandran, Aaron Pereira, Bernhard M. Weber, Nicolai Bechtel, Cornelia Riecke, Bernhard Brunner, Neal Y. Lii, Julian Klodmann, Anja Hellings, Katharina Hagmann, Gabriel Quere, Adrian S. Bauer, Marek Sierotowicz, Roberto Lampariello, Jörn Vogel, Alexander Dietrich, Daniel Leidner, Christian Ott, Gerd Hirzinger, Alin Albu-Schäffer

**Affiliations:** Institute of Robotics and Mechatronics, German Aerospace Center (DLR), Wessling, Germany

**Keywords:** telerobotics, model-augmented telemanipulation, shared control, shared autonomy, haptic constraints

## Abstract

Certain telerobotic applications, including telerobotics in space, pose particularly demanding challenges to both technology and humans. Traditional bilateral telemanipulation approaches often cannot be used in such applications due to technical and physical limitations such as long and varying delays, packet loss, and limited bandwidth, as well as high reliability, precision, and task duration requirements. In order to close this gap, we research model-augmented haptic telemanipulation (MATM) that uses two kinds of models: a remote model that enables shared autonomous functionality of the teleoperated robot, and a local model that aims to generate assistive augmented haptic feedback for the human operator. Several technological methods that form the backbone of the MATM approach have already been successfully demonstrated in accomplished telerobotic space missions. On this basis, we have applied our approach in more recent research to applications in the fields of orbital robotics, telesurgery, caregiving, and telenavigation. In the course of this work, we have advanced specific aspects of the approach that were of particular importance for each respective application, especially shared autonomy, and haptic augmentation. This overview paper discusses the MATM approach in detail, presents the latest research results of the various technologies encompassed within this approach, provides a retrospective of DLR's telerobotic space missions, demonstrates the broad application potential of MATM based on the aforementioned use cases, and outlines lessons learned and open challenges.

## 1. Introduction

Telerobotics is a powerful tool to combine the benefits of robotic manipulation with human mental abilities and manipulation strategies. Modern bilateral teleoperation systems provide haptic feedback that enables a human operator to perceive interaction forces and—more importantly—to intuitively control the forces applied by a teleoperated robot on its environment. This kind of feedback is crucial for delicate applications and tasks that comprise handling of fragile, dangerous, or expensive parts, or require high precision as it enables the operator to feel guiding structures or sliding on surfaces with limited forces. Such situations typically occur for applications in space, biochemical laboratories, or radiation environments. The latter was also the motivation for the development of many of the early telemanipulation systems that handled highly radioactive materials starting in the 1940s (cyberneticzoo.com, 2014). While these early systems were purely mechanically coupled, a revolution in telemanipulation occurred with the introduction of information technology (IT), which made it possible to electronically couple the haptic interaction device to the remote system. The major advantages of this innovation were (i) the ability to cover greater distances, (ii) a greater flexibility in control, (iii) a clearer presentation of forces, but above all (iv) a drastic reduction of apparent inertia. In addition to numerous incremental improvements in hardware and control approaches, there were a few other relatively new developments that significantly advanced the applicability and ease of use of telerobotics.

First, software-generated constraints that can limit the position or force of the haptic device or remote robot were introduced as so-called virtual fixtures (VFs; Rosenberg, 1993b). They guide the robot through a predefined desired path or restrict it from getting into a forbidden region of the workspace. Thus, VFs reduce the control freedom given to the operator while enhancing task accuracy and task completion time (Kang et al., 2004). They are also ideal for tasks requiring speed and precision while being repetitive in nature (Payandeh and Stanisic, 2002). Therefore, VFs are a great candidate for applications such as laparoscopic surgery, where they add an additional layer of safety and increase the surgeon's dexterity (Turro and Khatib, 2001). However, they have also proven to be highly beneficial for telemanipulation tasks with very long time delays (Xia et al., 2012).

Second, diverse forms of cooperation between operator and robot emerged, such as *supervisory control* (Ferrell and Sheridan, 1967; Sheridan, 1992) or *shared control* (Anderson, 1994). These approaches, subsumed under the term *shared autonomy*, aim at overcoming limitations of the operator that are due to the complexity of the robot or time delay between operator and robot by transferring some workload to the robot. Embedded into this concept, shared control refers to a continuous blend of human and robot control, ranging from safeguarding techniques (Fong et al., 2001), where the robot validates the operator's input, to adaptive virtual fixtures (Aarno et al., 2005), that support the operator in achieving predicted goals. Supervisory control, on the other hand, refers to an intermittent programming of the robot while the robot engages in a closed-loop interaction with its environment.

Third, model-mediated telemanipulation (MMT) was introduced where the user interacts with a local haptically rendered model estimate of the remote environment that is constantly updated, instead of being directly coupled to a teleoperated robot (Hannaford, 1989; Mitra and Niemeyer, 2008). The closed loop controller gets split into two control loops on either side of the communication channel, i.e., the haptic device and the remote robot side. Such an architecture reduces the conservatism while maintaining stability for arbitrary time delays. MMT has also been extended to multi-operator multi-robot systems to enhance performance compared to the classical bilateral approach (Passenberg et al., 2010). Despite its advantages, MMT has a few unresolved challenges. One of which is the unstable haptic rendering on the operator side during drastic changes in the updated local model. Another, and perhaps more significant, hurdle is the environment modeling. A model mismatch can result in transmitting dangerous position information for the robot to follow, which can end up with the robot exerting high forces and thereby damaging itself and the remote environment (Xu et al., 2016). To this end, reinforcement learning has recently been integrated into the concept of MMT in order to adapt to new environmental conditions and to cope with high uncertainties (Beik-Mohammadi et al., 2020).

While stability is not an issue in an ideal system without delays and with unlimited communication bandwidth, real-world scenarios, especially those with communication over long distances, pose additional challenges in terms of control. To this end, bilateral control approaches have been continuously evolved in parallel to the aforementioned developments, and today enable haptic telemanipulation via communication including time delays of several seconds (Panzirsch et al., 2020a). Although such approaches can guarantee stable operation, telemanipulation with such significant delay still remains demanding for the operator, and a more powerful approach facilitating the task would be useful.

**Figure 1:**
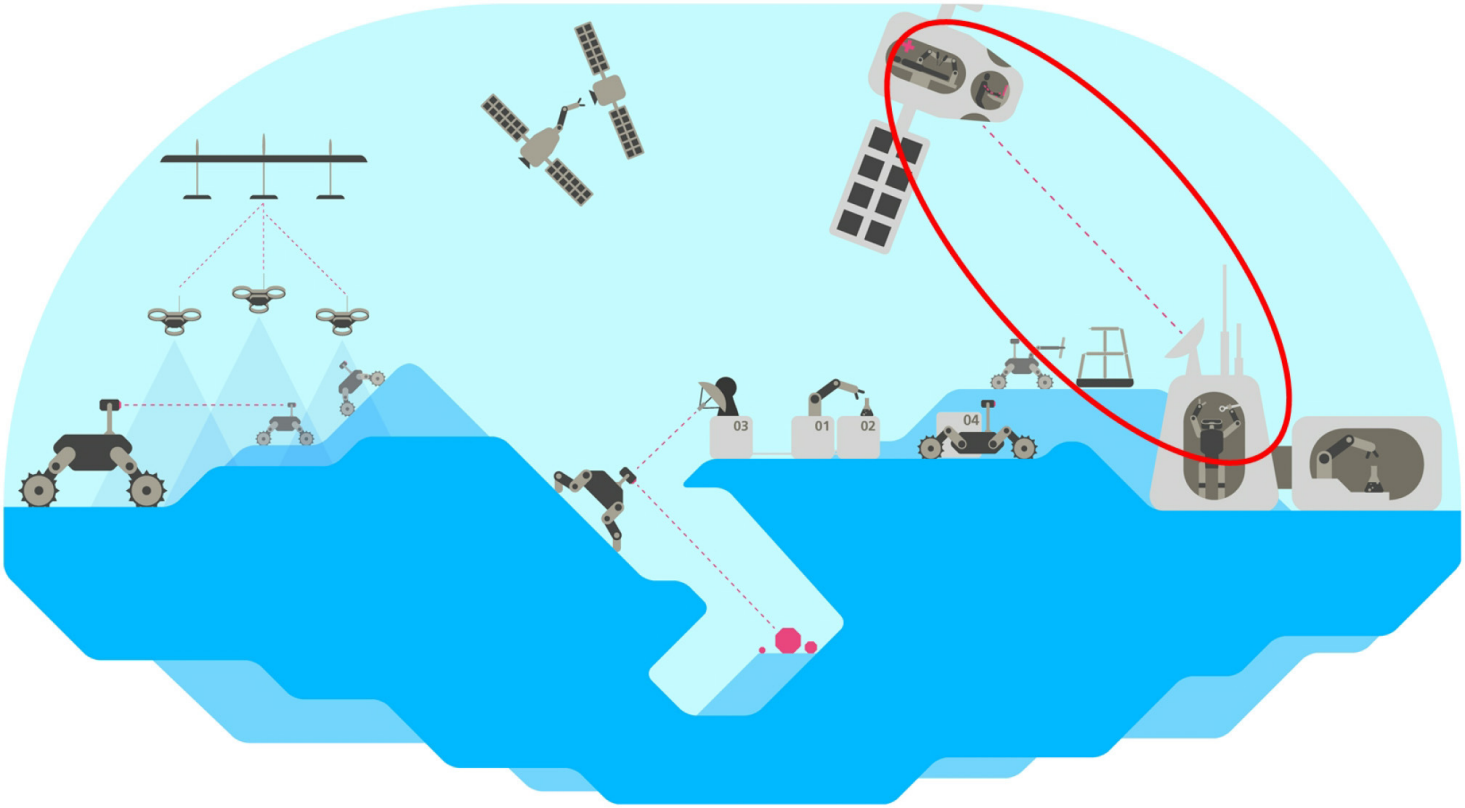
Illustration of DLR's space robot vision. Teleoperation is a key topic of DLR's long-term research endeavors for robot applications on celestial bodies and is illustrated by the example of telemanipulation of a humanoid robot from a spacecraft. While the number of tasks that robots can perform autonomously is steadily increasing, teleoperation will still be required over the next few years or decades for situations where autonomy fails.

One of the main research interests at DLR is to enable robots to operate in orbit and on the surface of celestial bodies and to perform exploration or construction tasks there. Figure 1 illustrates this vision and shows a spectrum of robotic systems to realize this goal. However, since robots are currently not able to operate fully autonomously, telerobotics is key to achieve this goal. The robots on the surface can be operated either from Earth or from a spacecraft, depending on the distance and the availability of a spacecraft.

This overview paper presents the model-augmented haptic telemanipulation (MATM) approach as a promising solution for such a telerobotic scenario. This approach uses two kinds of models, a remote model to enable shared autonomous functionality of the teleoperated robot and a local model to generate assistive augmented haptic feedback for the human operator. The forces that are displayed to the operator are a combination of augmented forces from the local model and forces resulting from interaction between the robot and the distant environment. The remote model is an environmental model of the remote environment to enable shared autonomy functionality to the teleoperated robot. The MATM approach can be considered as generalization of MMT, where the user interacts with a local model that acts as medium between the haptic device and the teleoperated robot. Yet, MATM has two major differences, i.e., the feedback to the human is a combination of real and augmented virtual feedback, and a remote model is introduced to enable shared autonomy of the remote robot.

**Figure 2:**
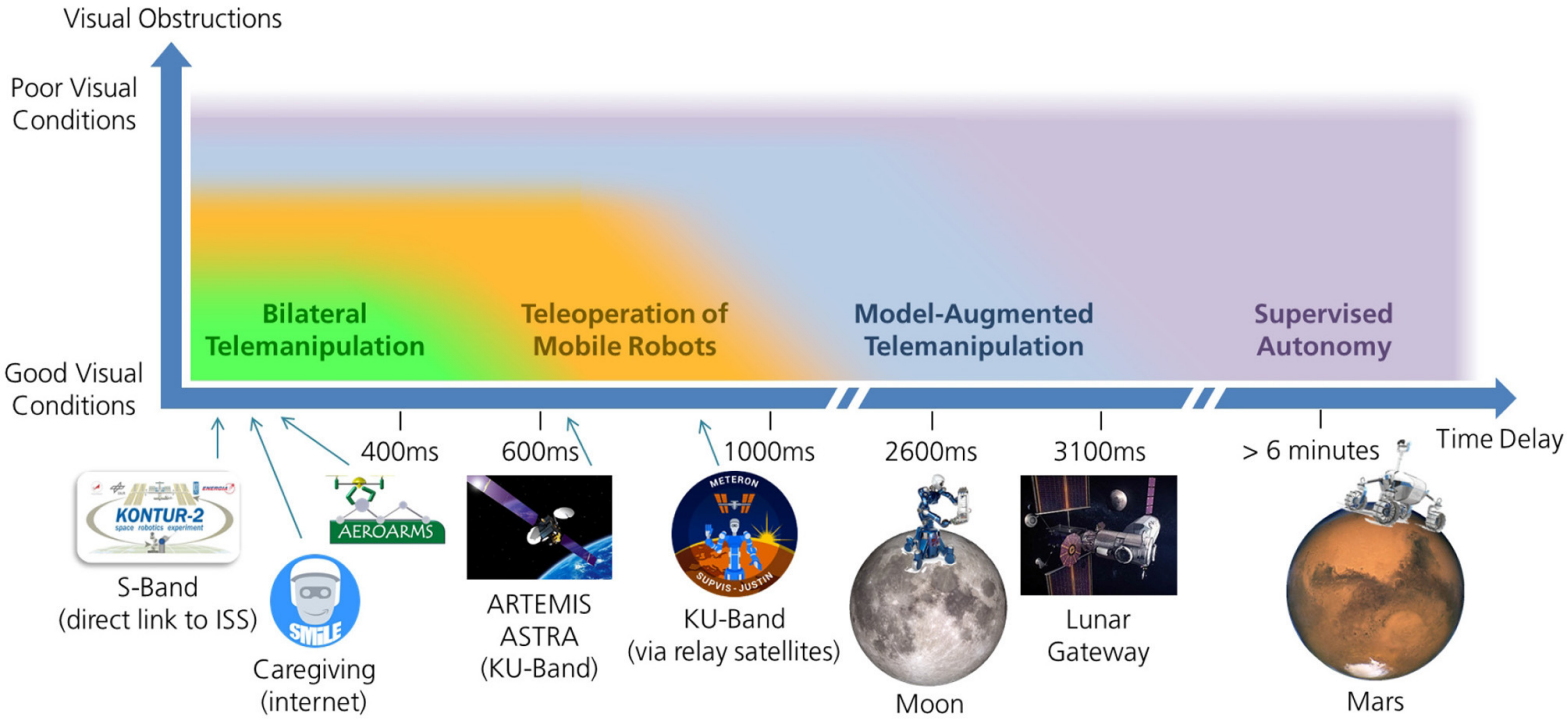
Schematic diagram that illustrates up to which time delay and under which visual conditions different telerobotic concepts can be applied. It also shows the delays that occurred for selected missions from section 3 and use cases from section 4 (credit for photos of the Moon, the Mars, and the gateway: NASA).

MATM can be regarded as an intermediate step toward supervised and fully autonomous manipulation. Figure 2 illustrates how this approach differentiates from classical bilateral telemanipulation, telenavigation of mobile robots, and supervised autonomy in terms of time delay and visual feedback quality. With increasing levels of support and autonomy, higher delays can be dealt with and visual quality demands decrease. The figure also shows the delays that occurred in some of the missions and use cases described in this paper.

The paper first presents MATM in detail and provides a state-of-the-art research overview in the underlying technologies used (section 2). Second, it gives a historical overview of the robotic space missions that were the main driving force behind this technology and highlights which aspects of MATM were advanced in each mission (section 3). Third, it discusses the potential and limitations of MATM based on use cases in different applications (section 4). In addition, the paper is also intended to serve as a reference work and therefore contains references to key publications that provide further details on specific aspects of the respective technology, mission, or use case.

## 2. Model-Augmented Telemanipulation

**Figure 3:**
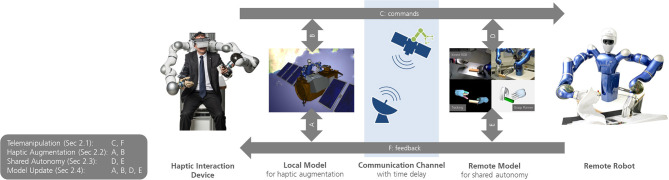
Illustration of the control scheme of MATM. The local and the remote model can both read and modify (or augment) the commands to the remote robot and the feedback to the operator. Certain methods and situations demand such full signal access, as explained in the respective subsections.

While in classical telemanipulation the operator is coupled to a remote robot via a haptic device, we aim to reach improved performance, efficiency, and ease of use during demanding telemanipulation tasks by means of two models that generate augmented feedback to the human operator and support the movements of the remote robot. Figure 3 schematically depicts this MATM approach and illustrates the elements that play a key role in it. The haptic interaction device acts as an input and output interface for the human operator and provides haptic force feedback. The remote robot is telemanipulated by the human operator and is intended to execute the desired commands in a remote environment. The communication channel connecting the two systems can cause a significant delay due to long transmission distances or limitations in the communication infrastructure. On each side of the channel, a model supports the movements or augments feedback, respectively. The following subsections describe the most important challenges in detail and outline our proposed solution. The applications of the methods described in this section along with its project or use case description will be presented in sections 3 and 4.

### 2.1. Telemanipulation Under Time Delay

Traditional bilateral control approaches, such as Lawrence's well-known 4-channel architecture (Lawrence, 1993), enable telemanipulation with force feedback. For space applications, other factors need to be considered, such as motion (Onal and Sitti, 2009) and force scaling (Goldfarb, 1998), which address the differences in precision requirements and is used for training purposes, or indexing (Johnsen and Corliss, 1971) which is a displacement technique to avoid reaching the workspace limits of the haptic device (Hagn et al., 2010). Most importantly, control approaches require considering the time delay in the communication channel that originate due to the huge distances between the operator and remote robot, which can have severe destabilizing consequences.

Extensive research has been carried out toward addressing the issue of stability for delayed bilateral teleoperation system, of which passivity-based methods are widely accepted and recognized due to their robustness and ease of applicability to any linear or nonlinear system independent of their model parameters. The Time Domain Passivity Approach (TDPA; Ryu et al., 2010) has garnered attention for being robust to variable delay and for being the least conservative of the passivity-based approaches. A novel 4-channel architecture using TDPA was implemented and tested in a real space experiment where the cosmonauts aboard the International Space Station (ISS) stably teleoperated a manipulator arm with two degrees of freedom (DoF) on Earth despite the inherent time delay (Artigas et al., 2016b). Nevertheless, TDPA too suffers from delay-dependent position drift and high frequency force oscillations. Therefore, some enhanced methods were proposed recently to remove this position mismatch between the haptic interaction device and remote robot while improving force transparency and enhancing the task performance (Coelho et al., 2018; Singh et al., 2018, 2019a; Panzirsch et al., 2020b).

Although position drift is an undesired phenomenon in telemanipulation, the authors of Panzirsch et al. (2020a) use it to their advantage to achieve a safe robot–environment interaction by using measured force feedback for the TDPA energy observations. Experimental validation for tasks such as slide & plug-in and pick & place were carried out safely and with a force feedback of sufficient quality, even with time delay of up to 3 s. This control algorithm was also extended for delayed telenavigation, where fictitious forces were generated by a set of “predictive” polygons, implemented in the driving direction of a mobile robot, overlapping with the objects in a depth data map (Sierotowicz et al., 2020).

Almost all of the state-of-the-art bilateral teleoperation controllers are implemented on both sides of the communication channel, i.e., on the local and the remote side. It would be advantageous if the stabilizing controller is implemented on either side of the communication channel, i.e., on the local side or on the remote side, as this would reduce the reliability on communication bandwidth and therefore diminish the effects of packet delay, loss, and jitter. This was recently achieved by the proxy-based controller (Singh et al., 2020) that is only implemented on the local side. Experimental results showed enhanced position synchronization and realistic impedance matching for a communication suffering from unknown time-varying delays of up to 2 s, and interacting with an active environment.

The above methods form the backbone of stable bilateral control even for communication that includes a delay of several seconds. On this basis, haptic augmentation and shared autonomy can enrich the telemanipulation framework as explained in the subsequent sections.

### 2.2. Haptic Augmentation

Haptic augmentation is the blending of the feedback from a remote robot with the feedback from a model. This haptic feedback is augmented to the haptic device so that it can be perceived by the operator and provide support during telemanipulation. While for many telemanipulation systems with negligible communication delay a distinction between local and remote model does not play a role, the situation is different for space applications in which communication delay affects the telemanipulation. Normally this feedback is implemented on the local model in order to obtain haptic support without delay. In some applications, such augmentation is also fed to the remote robot to achieve a more direct reaction and higher precision in manipulations tasks (this signal path is represented by the bi-directional arrow B in Figure 3).

A standard tool in haptics for generating such feedback are *haptic constraints*—also denoted as *virtual fixtures*. The concept of virtual fixtures was introduced by Rosenberg (1992) to support the operator during a telemanipulation task and was also evaluated for time-delayed systems (Rosenberg, 1993a). Virtual fixtures are control algorithms which regulate manipulator motion, surveyed in Bowyer et al. (2013). They are typically employed to support or guide the operator for high precision tasks, avoid critical regions in which the remote robot could cause some damage, and avoid running into robotic constraints such as workspace limits or singularities.

To enable more general geometries for virtual fixtures, haptic algorithms can be used instead of geometric primitives. A prominent example of such an algorithm is the *Voxmap PointShell Algorithm* that uses volumetric data structures and is able to compute collision forces at haptic rates (1 kHz) even for extremely complex geometries in multi-object simulations (McNeely et al., 2006; Sagardia, 2019). Such an algorithm can also be combined with a physics-engine to include physical phenomena in the simulation (Sagardia et al., 2014). This capability is very useful for telemanipulation to predict object movements and poses and thus counteract the effects of time delays.

The concept of augmented haptics can also be applied for bimanual telemanipulation tasks or multilateral teleoperation in which more than one haptic device or remote robot is used. For instance, if a high precision in orientation is demanded, two haptic devices can be coupled with a virtual rigid link to create an additional virtual grasping point that helps the operator to precisely set orientations (Panzirsch et al., 2018a). In cooperative tasks, where two operators jointly manipulate an object, knowing the intention of the respective other operator would be useful. Providing haptic information about the collaborating operator's intention is faster (force = acceleration) than on the visual/audio channel (velocity information). This concept was already evaluated in a delicate experiment with flexible objects involving the ISS (Panzirsch et al., 2017). In this experiment, the intention was measured by force sensors at the two haptic devices.

A challenge in this task is to differentiate between the feedback from intention and from the remote robot. In general, the operator should be able to distinguish between real and extended haptic feedback. One approach to achieve this is to apply a drastically higher stiffness for the amplified haptic feedback than for the one from the remote environment, which is feasible because the signals of the local path are not affected by the time delay of the communication channel (Hulin et al., 2013; Singh et al., 2019b). Another open question is how to best parameterize and distribute haptic augmentation between the local and the remote model. Future theoretical investigations and user studies should address this topic.

Feedback similar to haptic augmentation may also be implemented directly on the remote side and thus support the remote robot's movements without having to send commands over the communication channel first, making it faster and more precise compared to using a local model. This kind of model-based support of the remote robot belongs to the field of shared autonomy, which is discussed in the subsequent section.

### 2.3. Shared Autonomy

Commanding robots is a highly demanding, tedious task for humans. This is partially because of the sheer number of degrees of freedom that need to be orchestrated, partially because of time delays that cause adoption of the move-and-wait strategy (Ferrell, 1965). *Shared autonomy* (also known as *mixed initiative interaction*) is an umbrella term subsuming multiple techniques that aim at reducing the workload of the operator by delegating some of the control to the robot (Goodrich et al., 2013). Examples of shared autonomy are *supervised control*, where the operator commands the robot intermittently with high-level tasks while the robot engages in a closed-loop interaction with the environment, and *shared control*, where continuous input from the user is processed by the robot in order to validate, augment, or map it to higher dimensions.

In the MATM approach, we use these techniques to support the operator while performing a telemanipulation task. In shared control, the robot may relieve the operator by taking over certain subtasks of the robot. An easy-to-understand example is to constrain the orientation of a manipulated object (Quere et al., 2020). To achieve such support, the methods of the previous section on haptic augmentation can be used and applied on the remote robot. The advantage compared over applying haptic augmentation to the haptic device is higher precision and faster reaction (without the delay of the communication channel). In addition, the shared control algorithm can take control of non-telemanipulated robot parts or joints. An example is automatic positioning of robot hand fingers to establish stable grasp (Hertkorn, 2016).

In a mixed initiative shared control approach, the weighted sum of the commands (positions/forces/torques etc.) from both agents, namely the human operator and the autonomous system, is given to the remote robot as the final command signal (Musić and Hirche, 2017). The weights for the individual commands are called task or authority allocation factors (Parasuraman and Riley, 1997). These factors can be fixed (Panzirsch et al., 2018a), or time varying to account for certain situation changes (Inagaki, 2003). In a recent publication, we developed a novel time varying approach, where the authority is shifted from the autonomous system to the human operator based on real measurement noise (Balachandran et al., 2020a) using Bayesian filters. This means that in case the autonomous system is not able to complete the task at hand due to bad measurements, the human operator is asked for intervention and to implement corrective measures to complete the task. If and when the sensor measurement quality improves, the control authority is smoothly given back to the autonomous system. This reduces the physical and mental efforts demanded from the operator as he has to intervene if and only when the autonomous system has low confidence in its own task completion ability.

Although more robust, fixed authority allocation-based shared control limits possibilities of human intervention in case of failure of autonomy. On the other hand, adaptive allocation factors are more robust against autonomy failures but are sensitive to the probabilistic filters' convergence. Further improvements can be made to optimize the mixed-initiative approach by combining confidence factors from both autonomy and the human operator, availing possibilities offered by artificial intelligence and machine learning.

While shared control depends on continual input from the user, supervised control can deal with intermittent input and is thus suitable for commanding multiple robots. We apply supervised control in a two-step approach. First, the user specifies a goal in an intuitive user interface (UI) (Birkenkampf et al., 2014), which is then translated into *Planning Domain Definition Language* (Ghallab et al., 1998). Second, the robot uses its local autonomy to reach the goal without any further need of user intervention. The local autonomy is based on *action templates*, which store the symbolic and geometric descriptions for manipulation instructions. The robot creates a plan to reach the specified goal based on the symbolic description in the action template headers. Robot-specific geometric reasoning modules then evaluate the geometrical descriptions of the respective action templates. In case of an error, the planner first assesses possible alternative geometric solutions before initiating backtracking to explore different solutions by re-evaluating previous actions. The procedure is described in detail in Leidner (2019) and the approach has been validated in multiple experimental sessions with astronauts on board the ISS (Schmaus et al., 2019). We also extended this approach to probabilistic domains where actions can fail (Bauer et al., 2020). This allows operators to choose between plans that reach the goal with different likelihood. Sometimes, operators might be willing to trade success probability for completion time, number of steps of the plan, or possible side effects.

Ongoing work focuses on how to switch from teleoperation to supervised control, which requires to update the world model according to the changes induced by the teleoperated robot. The challenges that arise during model updates and their respective solutions are the subject of the following section.

### 2.4. Model Update

The model update represents the updating of the data of the local and the remote model as well as the synchronization between these two models. Two challenges arise directly from this task. First, how can the models be synchronized even though the data of the models may be in a different structure or representation? Second, how can stability be established despite the fact that the updating process is highly nonlinear, especially in case of time delay, jitter, and packet loss?

In the case of supervised control, model update translates to keeping the local model (that is shared between robot and operator) in synchronization with the environment the robot is acting in. This includes detection and localization of objects, but also inference of the symbolic state. Both geometric and symbolic information are needed by the user interface for providing the user with possible actions and by the robot in order to execute those actions. A viable and pragmatic solution for this is a shared knowledge base that stores the object information and provides it to both modules in order to create a knowledge common ground. Part of this knowledge base can be geometric models, available action templates, symbolic state, and pose of the objects. In our implementation, object detection and localization are performed according to Sundermeyer et al. (2018). The symbolic state of the environment is evaluated based on a digital twin of the robot and the environment in simulation as described in Bauer et al. (2018).

In order to tackle the second challenge, i.e., the stability of the overall system, we research a novel control framework. The challenges of the proposed framework in terms of closed-loop stability are the fusion of different force feedback channels with computed, measured, or fictitious forces and the design of the reference position for the devices. Those challenges also include the model update, which represents a highly nonlinear functionality especially in the presence of time delay, jitter, and packet loss, making it a potential source of instability.

The energy-based passivity principle represents a highly modular method to assure absolute stability of complex closed-loop systems since the passivity of submodules can assure the passivity of the overall system. Thus, different modules as the force feedback channel or the haptic augmentation and shared autonomy functionalities (compare Figure 3) can be designed and activated or deactivated, respectively, in a highly adaptive and modular manner. The fusion of different force commands to the haptic input device and remote robot can be passively designed with the help of power control units as earlier presented for multilateral telemanipulation (Panzirsch et al., 2013), telenavigation (Panzirsch et al., 2018b), and haptic augmentation (Panzirsch et al., 2017, 2018a). Exemplary, the haptic augmentation and shared autonomy modules based on local and remote models can be modeled as 1-port subsystems, which can be designed to be intrinsically passive (Weber Martins et al., 2018) or, alternatively, passivity controllers can assure the passivity of the modules including model updates as proposed in Xu et al. (2015) and Panzirsch et al. (2018b).

The modularity of the passivity concept simplifies combining independently developed passive modules, since no complex stability analyses of the overall control loops are required. The remaining challenge is the passive design of prospective haptic augmentation and shared autonomy features. It should be noted, though, that passivity is in general not more conservative than the popular Lyapunov stability criterion, especially since passivity does not necessarily have to be ensured in the frequency domain, but can be guaranteed in a highly adaptive manner in the time domain.

## 3. Past Telerobotic Space Missions—Prior Milestones on the Way Toward Model-Augmented Teleoperation

**Figure 4:**
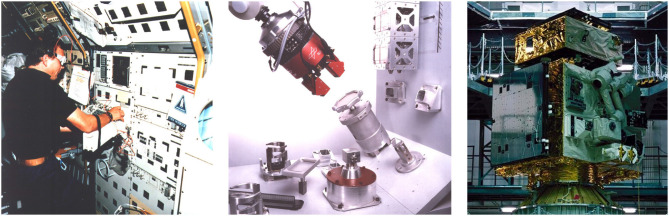
The astronaut Hans Schlegel inside the Space Shuttle Columbia (**left**, credit: NASA) controls the robotic gripper of the ROTEX experiment **(middle)**. The chaser satellite of the ETS-VII mission was equipped with a robot arm (**right**, credit: NASA).

The starting signal for DLR's telerobotic space missions was given in 1993 with ROTEX (Figure 4). Since then, DLR has contributed to several telerobotic space missions in cooperation with various space agencies, in particular ESA, ROSCOSMOS, and JAXA. The most significant missions for the MATM approach are briefly described in this section. In contrast to a purely historical overview on our telerobotic missions (Artigas and Hirzinger, 2016), this section is intended to relate to the MATM approach and to highlight the specific impact of our past missions, to synthesize the lessons learned, and to point out the challenges ahead.

### 3.1. Model Predictive Teleoperation—ROTEX and ETS-VII

The first space robotics experiment performed by DLR was the ROTEX experiment (Hirzinger et al., 1993) during the D2 mission in 1993 on board the Space Shuttle Columbia. A multisensory robot inside the spacecraft successfully worked in four operational modes, i.e.,

- automatic (preprogramming on ground, reprogramming from ground),
- teleoperation on board (astronauts using stereo-TV-monitor),
- teleoperation from ground (using predictive computer graphics) via human operators and machine intelligence as well,
- tele-sensor-programming (learning by showing in a completely simulated world on ground including sensory perception with sensor-based execution later on board).

**Figure 5:**
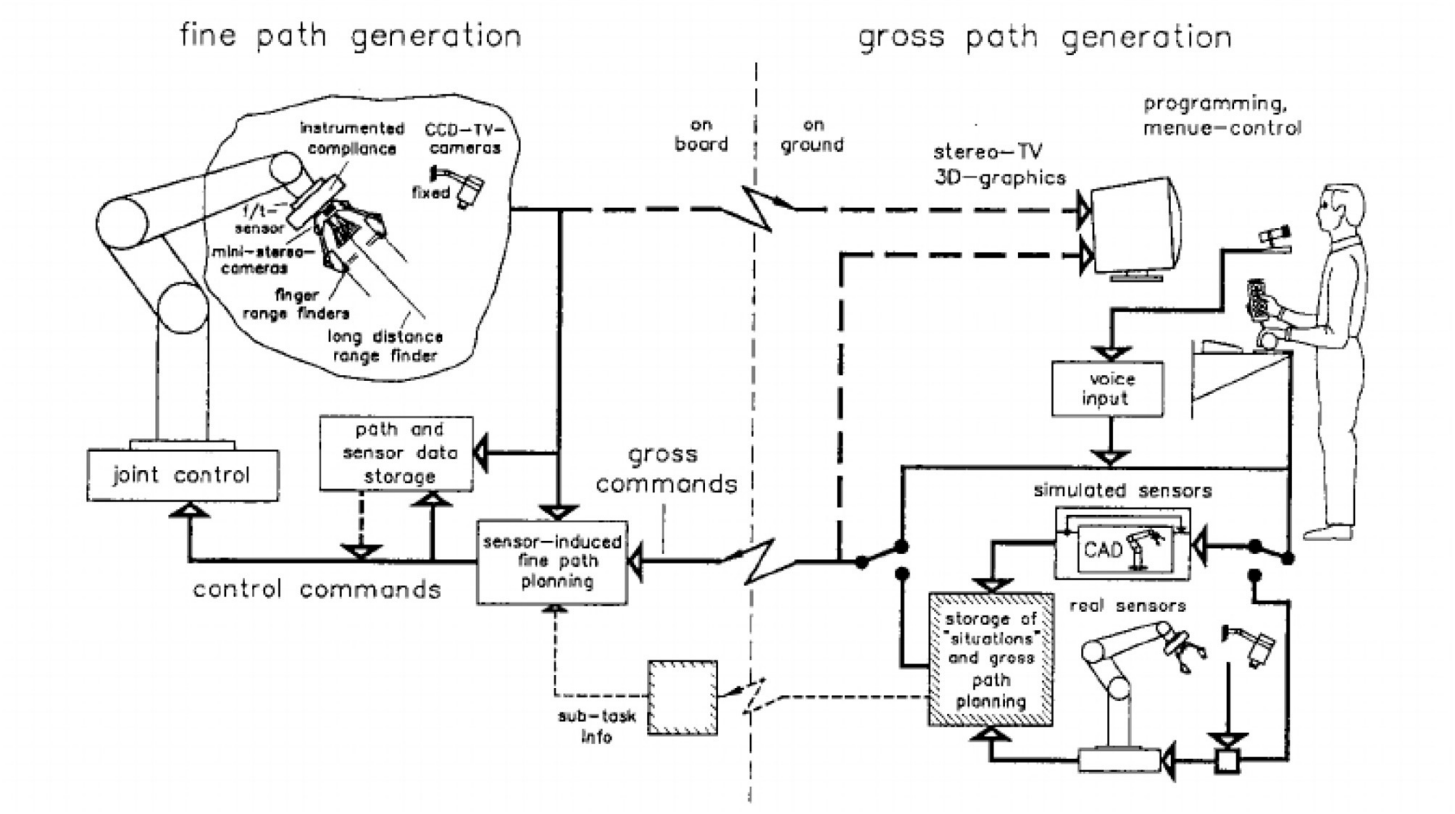
The overall loop structures for the sensor-based telerobotic concept of the ROTEX experiment (Hirzinger et al., 1993). [©1993 IEEE. Reprinted, with permission, from (Hirzinger et al., 1993)].

The main control concept behind all these modes was a shared autonomy approach, which includes shared control as well as shared intelligence, based on local autonomy loops on board. Figure 5 shows the overall loop structures for the sensor-based telerobotic concept.

Due to the large time delays of up to 7 s that were involved during operation from ground, there was no haptic feedback in the ROTEX experiment. Instead, the human operator was enclosed in the feedback loop via stereovision and 3-D graphics on a very high level but with low bandwidth, while the low-level sensory loops were closed directly at the robot on board with high bandwidth.

To handle the large time delay, ROTEX used a predictive computer graphics approach, which seems to be the only way to overcome this problem. A human operator at the remote workstation gave robot commands by looking at a *predicted* graphics model of the robot. The control commands issued to this instantaneously reacting robot simulator are sent to the remote robot as well using the time-delayed transmission links.

Complex tasks were split up into elemental moves, represented by a certain configuration, which allows the simulated (as well as the real) robot to refine the gross commands autonomously. We introduced the term *tele-sensor-programming* that means the robot is graphically guided through the task (off-line on ground), storing not only the relevant Cartesian poses of the gripper but also the corresponding nominal sensory patterns (graphically simulated) for later reference in the on-board execution phase.

In summary, this mode of tele-sensor-programming is a form of off-line-programming, which tries to overcome the well-known problems of conventional approaches, especially the fact that the simulated and the real world are not identical. But instead of calibrating the robot, tele-sensor-programming provides the real robot with simulated sensory data that refer to relative positions between the end-effector and the environment, thus compensating for any kind of inaccuracies in the absolute positions of the robot and the real world. Using the simulated sensor values during the programming phase can be seen as the first model-based teleoperation approach in space robotics.

A few years later in 1999, DLR got the chance to contribute with an own experiment (German ETS-VII Technology Experiment [GETEX]) to the Japanese ETS-VII mission, which was the first space robotics mission with a focus on on-orbit-servicing tasks. For DLR the participation was the first step to a big challenge in space robotics, i.e., the capturing and repair of a failed satellite, completely controlled remotely from ground. In that context, we performed two main tasks, first a series of dynamic experiments to verify our models of free-floating space robots and the identification of the dynamic model parameters; second—and this is the more interesting one in the field of telerobotics—a peg-in-hole experiment, using VR methods and a *vision-and-force* control scheme, by closing sensor control loops directly on board (force) and via ground communication (vision). Like in ROTEX we used the tele-sensor-programming approach to set the reference values for the visual servoing task, using some dedicated markers as image features, in a virtual environment for later usage in space. For that we developed an approach, which did not need any calibration, because it was only based on the sensor–actor relation: the desired Cartesian goal frame of the robot's tool center point was expressed only by the respective visual sensory pattern (Brunner et al., 1999).

### 3.2. Force-Feedback—ROKVISS and Kontur-2

**Figure 6:**
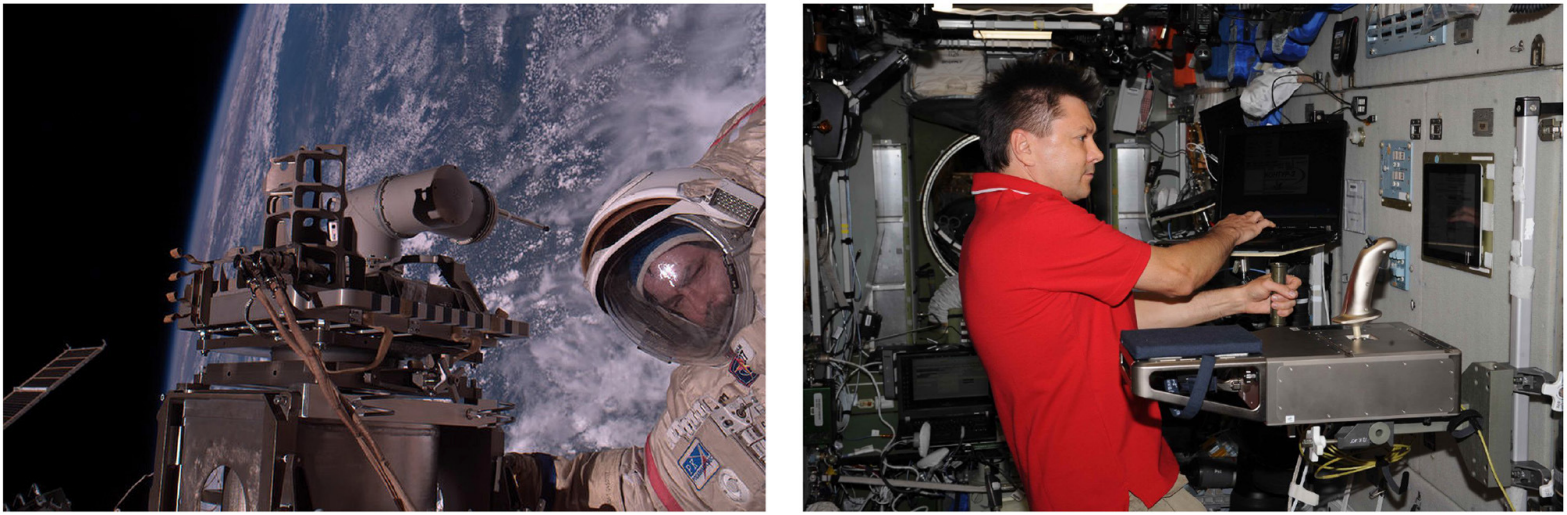
The ROKVISS system mounted on a platform on the outside of the ISS (**left**, credit: NASA). Cosmonaut Oleg Kononenko with the Kontur-2 joystick on board the ISS (**right**, credit: ROSCOSMOS/O. Kononenko).

Launched in 2005 and operated for nearly 5 years in space, the Robotics Component Verification Experiment on the ISS (ROKVISS) was a big success for two reasons: the first aim was the in-flight verification of highly integrated modular robotic joints (Figure 6, left), the second one the demonstration of different control modes, reaching from high system autonomy to force feedback teleoperation (telepresence mode). After ROTEX and GETEX, which did not cover any haptics, ROKVISS was designed to test and verify real telepresence operation using haptic and visual feedback at high data rates. For that the telepresence system of ROKVISS was equipped with

- a highly dynamic teleoperated robot including sensors and local intelligence,
- a high-bandwidth real time communication channel,
- an immersive multimodal human–machine interface.

All these components had to be connected by an advanced control concept, which combined shared autonomy and bilateral control of the teleoperated robot and guaranteed a synchronicity between the visual and haptic information. The human–machine interface played a major role for immersive telepresence. The operator should feel like being at the remote location. Therefore, DLR developed a new 2-DoF force-feedback joystick corresponding to the two joints of the manipulator in space. Providing the human operator with haptic feedback means to include the human into the control loop, i.e., the human arm was energetically coupled with the manipulator arm at the ISS. The stabilization of this coupled telemanipulation system was complicated due to the presence of time delay in the system (Hannaford and Ryu, 2002). An advantage of ROKVISS was that the communication delay was relatively small (10–30 ms) and predictable. This allowed to simulate additional time delays to test different control schemes and communication systems within a real space experiment (Preusche et al., 2006).

Space agencies are planning and working toward crewed lunar and planetary exploration missions to be realized within the next few decades. Sending astronauts directly to the surface of the celestial bodies is extremely dangerous and costly. Therefore, in an initial, cautious step, robots can be teleoperated from an orbital spacecraft to explore the surface, acquire samples, and construct habitats. To this end, DLR and the Russian space agency ROSCOSMOS collaborated on the Kontur-2 mission during the period 2012–2016.

The main goal of the Kontur-2 space mission was to test the feasibility of using force feedback teleoperation from a spacecraft in micro-gravity conditions and to telemanipulate robots on distant planets (Riecke et al., 2016). For this, the ISS was used as the spacecraft and the Earth as planet with a robot on its surface. It was therefore an inverted scenario compared to ROKVISS. To provide high fidelity force feedback to the cosmonauts, DLR developed a space qualified force feedback joystick, which was taken on board the ISS (Figure 6, right). A direct link over S band was used for communication between the ISS and Earth with short latency and ISS experiment windows. In spite of the short latency (10–30 ms round-trip delay), it was observed that the bilateral controller was unstable due to the closed control loop with force feedback.

To reduce the performance deterioration that comes as the trade-off while ensuring stability, a novel 4-channel architecture bilateral controller was developed with passivity observers and passivity controllers as explained in section 2.1. This 4-channel bilateral controller provided a stable and highly transparent teleoperation system in spite of the communication delays and data losses and was tested in both terrestrial set-up (for cosmonaut training) and for the real space mission (Artigas et al., 2016b). In addition to the single-operator single-robot teleoperation, further tests were conducted for cooperative grasping of objects by two users.

In the scenario, a cosmonaut on board the ISS and a second operator from ground (located at our project partner in Russia) teleoperated a dual arm robot at DLR to cooperatively handle a flexible sphere. In order to handle the sphere safely (without dropping it or pressing it with too high forces), the haptic intention augmentation approach explained in section 2.2 was tested and verified during the Kontur-2 mission (Panzirsch et al., 2017). It was learned that force feedback provided the cosmonaut with a more intuitive feeling of the robot-environment interaction with which he could modulate the interaction forces more accurately as desired.

A series of human factors experiments was conducted within the Kontur-2 space mission, investigating the benefits of force feedback under conditions of weightlessness. Cosmonauts teleoperated the ROKVISS robot from the ISS with DLR's force feedback joystick. Findings indicated that force feedback is indispensable for teleoperation tasks, although the terrestrial performance level could not be reached in weightlessness. Moreover, haptic support at the joystick (e.g., motion damping) has to be adjusted to be beneficial in weightlessness conditions and higher resistive forces should be avoided (Weber et al., 2019, 2020; Riecke et al., 2020).

### 3.3. Supervised Autonomy—METERON SUPVIS Justin

Space telerobotics based on haptic telepresence provides close, immersive coupling between the user and the robotic asset. However, it presents two drawbacks: short effective operation time due to user fatigue, and difficulty to scale up (Lii et al., 2018). METERON SUPVIS Justin was a mission to tackle these issues with a different approach to teleoperation with supervised autonomy, or shared autonomy. Rather than using the robot as a haptically coupled avatar for the user, the robots are utilized as intelligent robotic assets, or coworkers to be commanded at the task level.

Between 2017 and 2018, three ISS-Earth telerobotic experiment sessions were carried out with five NASA and ESA astronauts. For METERON SUPVIS Justin, an analog scenario of a Martian surface environment was implemented at DLR in Germany to be serviced by DLR's humanoid robot Rollin' Justin (Borst et al., 2007, 2009). ISS in turn takes on the role of the orbiting spacecraft, from where the astronaut commands the robots on simulated Martian surface.

To test the robot's ability to carry out an increasing catalog of tasks that could be expected in a space habitat or colony, the SOLar farm EXperimental (SOLEX) environment was developed and constructed at DLR in Oberpfaffenhofen, Germany. The SOLEX environment is equipped with a wide array of systems and devices including solar panels, smart payload units, and a lander, which allowed for the design of different mission scenarios to be carried out by the human–robot team (Bayer et al., 2019).

**Figure 7:**
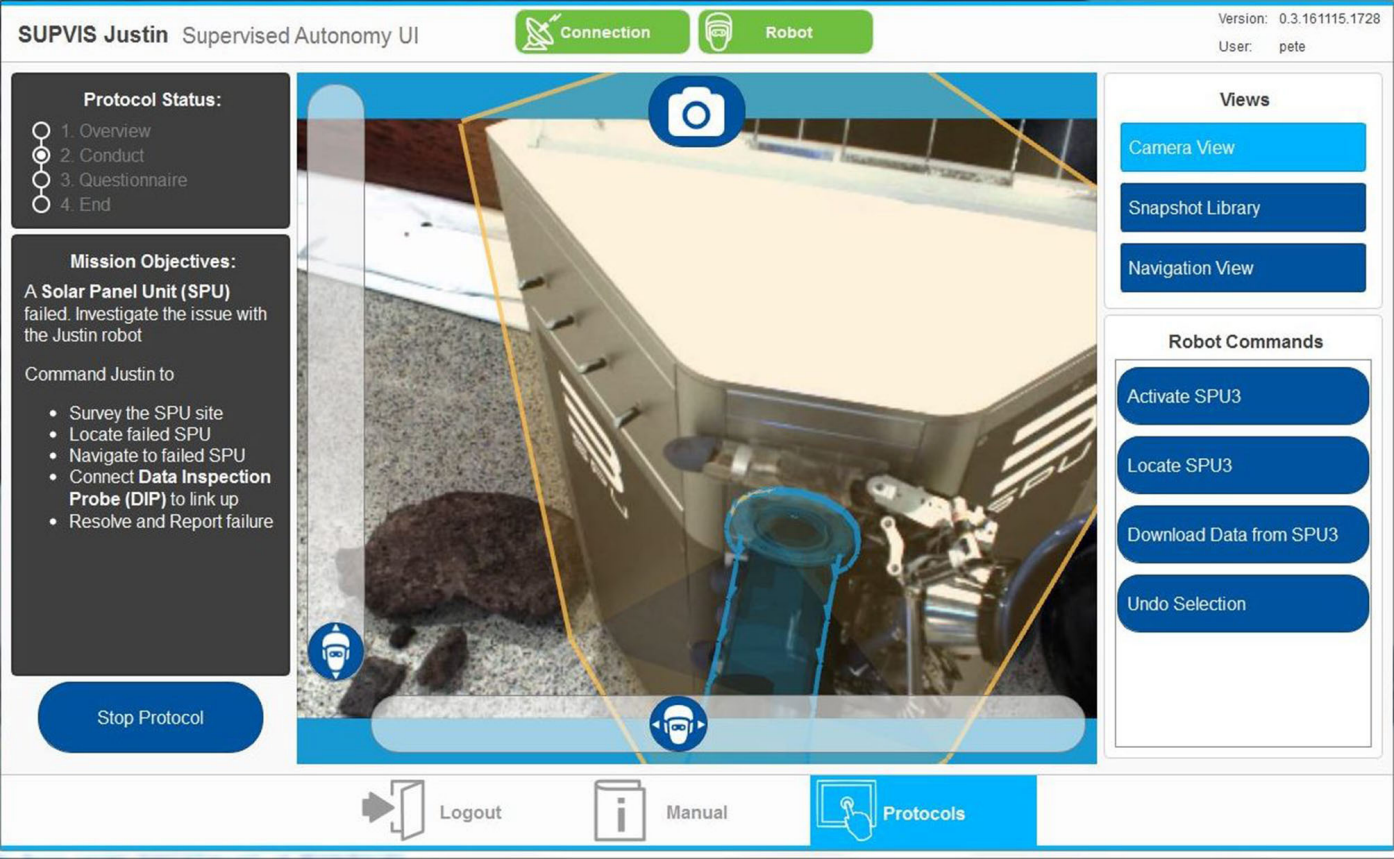
An example layout of the knowledge-driven intuitive tablet user interface on board the ISS (Lii et al., 2018).

Using action templates (Leidner, 2019) as described in section 2.3, Rollin' Justin carried out the task level commands provided by the astronaut by utilizing its local intelligence to process and execute lower level tasks. The knowledge-driven approach was also applied to the user interface design in the form of an intuitive touch screen tablet application (Schmaus et al., 2019). Implemented on a commercial off-the-shelf (COTS) tablet PC, the application provides the crew with vital information on the mission at hand, view from Justin's camera, and a dynamically updated list of relevant commands. This provides an uncluttered and intuitive user interface to command a highly complex robotic asset. Figure 7 shows the user interface on the tablet PC being commanded by the ISS crew.

**Figure 8:**
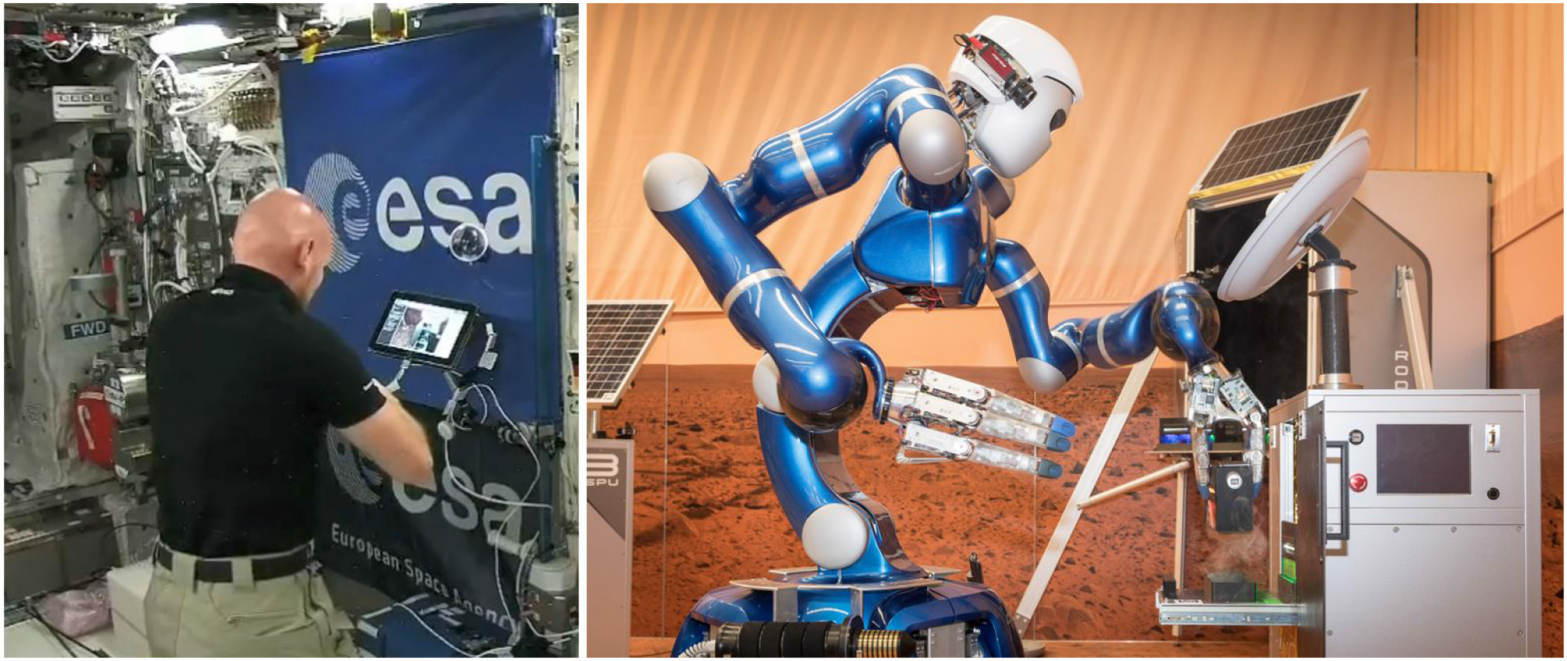
ESA astronaut Alexander Gerst on board the ISS (**left**, credit: ESA) commanding DLR's Rollin' Justin in the SOLEX environment to perform component retrieval and assembly tasks **(right)**.

Through three sessions, increasingly complex tasks were carried out: from service and inspection, to manual device adjustment and maintenance, concluding with a full set of component retrieval and assembly tasks. Figure 8 shows ESA astronaut Alexander Gerst performing component retrieval and assembly with Rollin' Justin. Thanks to the supervised autonomy approach, all participating ISS crew members not only were able to successfully complete all assigned tasks, their feedback also indicated that they would be able to handle working with larger robotic teams to perform more complex tasks with this approach.

### 3.4. Telenavigation—Analog-1

**Figure 9:**
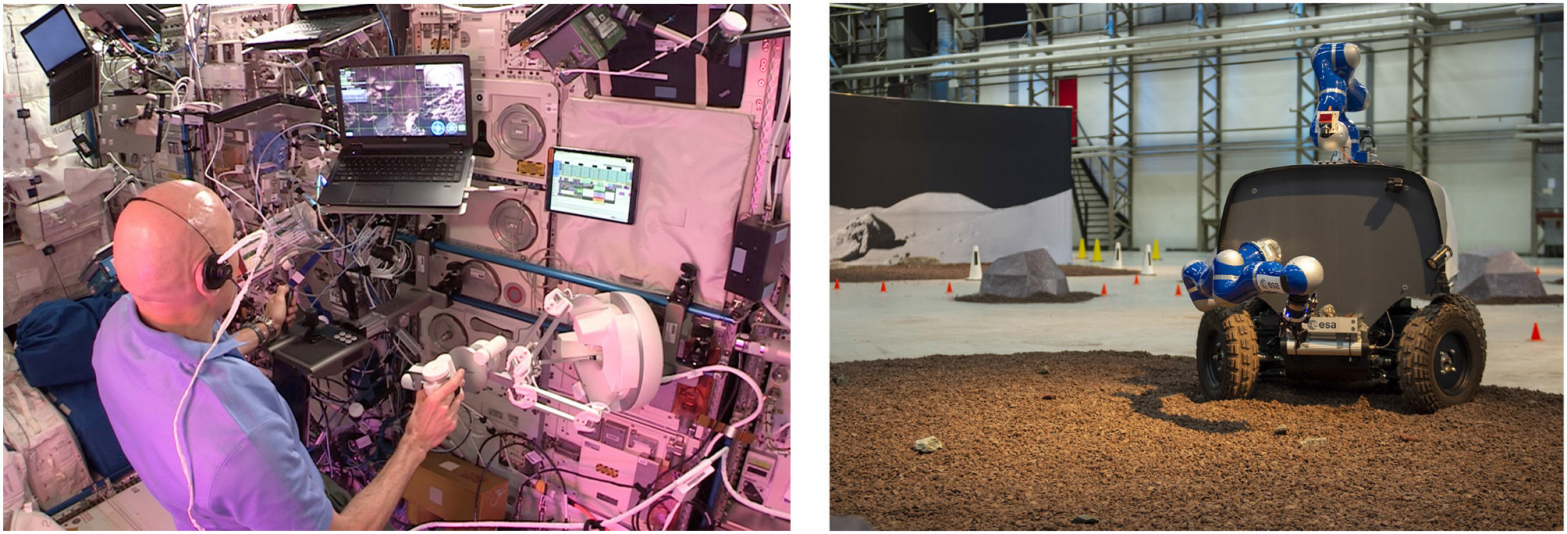
**(Left)** Astronaut Luca Parmitano used a haptic device and a joystick to control the robot arms and the mobile platform (credit: ESA). **(Right)** The Analog-1 mobile platform at a mocked-up geological sampling site (credit: ESA).

The Analog-1 mission (November 2019) tested geological sampling from orbit. It was intended to give insight into the feasibility of operating a robot on the surface of the moon by an astronaut aboard the Lunar Gateway, where communication latencies would be comparable to, or less than, those from ISS to ground (these were ≈850 ms in the K_u_ band link via relay satellites). In contrast to the SUPVIS-Justin experiment of the previous section, the unstructured environment and loosely defined tasks made supervised autonomy impractical. Hence, for the first time, full-DoF direct teleoperation with force feedback was tested from space to ground. The robot controlled from space was a mobile platform with two robotic manipulators and two cameras, shown in the right photo in Figure 9. The astronaut on the ISS drove the mobile platform to three geological sampling sites (mocked-up in a hangar in the Netherlands), investigated them and collected rock samples, all while in communication with geologists.

The astronaut's work station consisted of a laptop to display and interact with the user interface; a Sigma.7 haptic interface device from Force Dimension (modified by the company to be used in microgravity) to command position of the tool on the manipulator and receive force feedback; and an integrated joystick with keypad to drive the platform, move the cameras, and also interact with the user interface (see Figure 9, left). For the control, we used TDPA to deal with latency (described in section 2.1). Full details of the control are outside the scope of this paper.

The astronaut was able to command the robot stably, effectively, and intuitively. Despite the unstructured environment, it was clear from pre-trials with astronauts and astronaut trainers that certain maneuvers could also be automated, for example, the stowing of the rock. This begs the question of how to scale up and down levels of autonomy for different environments or tasks, with the same interface. Furthermore, possible uses of augmented reality were identified: to aid communication with scientists (during the experiment the astronaut benefited from a grid projected over the image), to aid driving under time delay (e.g., to show the projected path of the platform under the current steering angle) or in semi-autonomous driving, and to specify via points for the robot path on the camera image itself.

## 4. Case Studies

While space missions were our original motivation for research on the MATM approach, it is evident that numerous other applications can also benefit from this approach. In this section, six exemplary use cases are presented to illustrate the wide variety of potential applications that reach from orbital applications over terrestrial telemanipulation in caregiving and telesurgery to applications that involve driving and flying robotic systems. In each use case description, special emphasis is given to the specific challenges, technical solutions, and experience gained. In none of these use cases, we have exploited the full spectrum of MATM so far, but rather emphasized certain aspects that appeared to be of particular interest for the respective use case. These foci are indicated in parentheses in the section headings.

### 4.1. In-orbit Telemanipulation (Haptic Augmentation and Shared Control)

**Figure 10:**
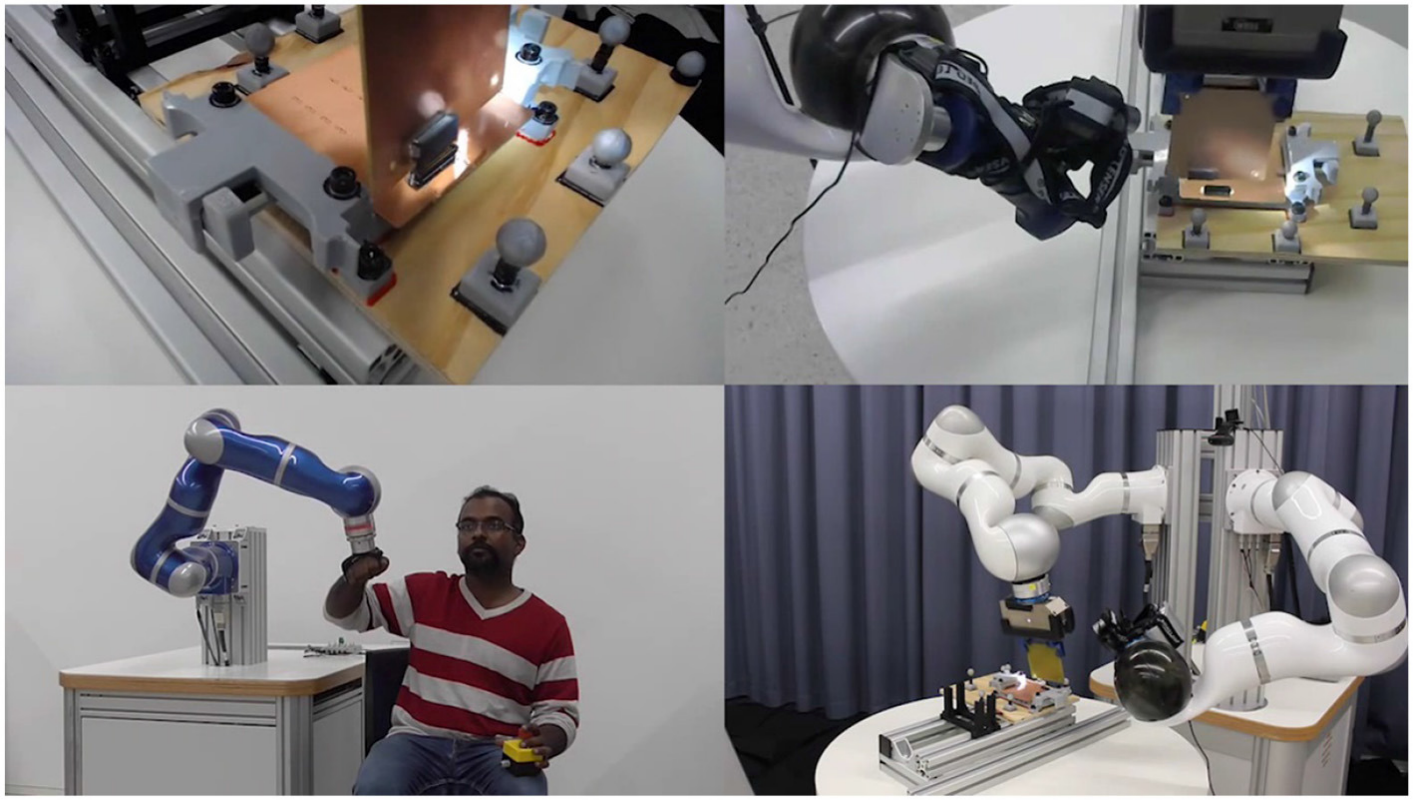
Demonstration of a telerobotic high-precision assembly task of an electrical connector of CubeSats (Weber Martins et al., 2018).

To reduce the cost and payload volume of satellites launches, the assembly of satellites may be realized in in-orbit factories (Spaceflight, 2020). Although manufacturing in the ISS has been recently tested with 3D printing (Napoli and Kugler, 2017), robotic assembly of satellites has not been done yet. To this end, an on-ground feasibility study was conducted within the framework of the Space Factory 4.0 project (Weber Martins et al., 2018) for the robotic assembly of CubeSats (Figure 10). *Space Factory 4.0* aimed at developing a bilateral controller, which allows for teleoperation of the assembly robot by a human operator using an HMI device, providing force feedback with the support of virtual fixtures, which in the control scheme of Figure 3, are elements of the remote model. The virtual fixtures are dynamic and are placed on the desired point by a vision-based tracking system. The final control architecture was based on a mixed-initiative approach (see section 2.3), where the final control commands to the remote robot was a weighted sum of control commands from the teleoperator and the vision-based autonomy (virtual fixtures) with fixed authority allocation factors (Panzirsch et al., 2018a).

In order to reduce the physical effort demanded from the operator while telemanipulating the remote robot using the robot-based haptic device with its high inertia, a local explicit force controller was used to match the forces measured at the human–haptic device interface to the force measured at the remote robot's end effector (Balachandran et al., 2020b). This reduced the perceived inertia of the haptic device by the operator, during free motion of the robot, and also increased the transparency during robot–environment interaction. In addition to the feedback of the measured forces from the robot's end-effector, additional feedback was provided to the operator via the forces generated by the virtual fixtures. This supported the operator in gaining a better impression of the relative motion of the robot with respect to the workpiece and the target position and orientation. In a pilot study, it was found that such supportive feedback reduced the required completion time for an assembly task of CubeSat parts that required a high degree of precision (Weber Martins et al., 2018).

In spite of these benefits introduced by this mixed-initiative-based shared control architecture, it was observed that if the virtual fixtures were wrongly placed during certain scenarios then the tracking system produced noisy measurements. Due to the fixed authority allocation factors that were tuned before-hand, the operator had to produce more physical effort to intervene and force the robot against the virtual fixtures. Future works include applying the adaptive shared control approach (as in Balachandran et al., 2020a) with possibilities for human intervention with more ease along with optimal placement of the virtual fixtures using artificial intelligence and machine learning.

### 4.2. Caregiving (Shared Autonomy and Seamless Autonomy Activation)

The demographic change in most industrial countries will pose major challenges to national health-care systems and the society to be faced within the next decades. While the number of people requiring assistance and caregiving is continuously growing, the number of caregivers is not keeping up with that demand. Robotic systems can potentially contribute to bridge this gap between demand and supply (Riek, 2017). Only recently, various robotic systems were brought to market for this purpose (Ackerman, 2018; Gupta et al., 2019; Mišeikis et al., 2020). Such robots should be able to take over logistical tasks or assist in care or daily life. Besides technical aspects, also the simplicity and empathy in interactions are highly relevant for these assistance tasks (Pepito et al., 2020).

**Figure 11:**
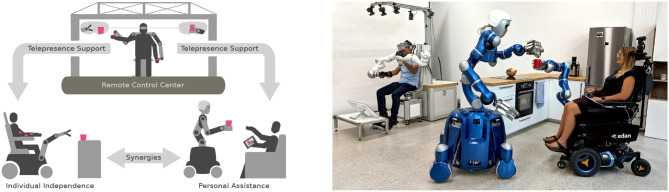
**(Left)** Concept of the caregiving ecosystem *SMiLE*. **(Right)** Exemplary implementation (Vogel et al., 2020). [©2020 IEEE. Reprinted, with permission, from (Vogel et al., 2020)].

To mitigate the demographic challenges, the prototypical *SMiLE*^1^ ecosystem has been developed as a holistic concept for robotic assistance in caregiving (Vogel et al., 2020). This ecosystem offers a variety of control modes and autonomy levels to meet the actual application at hand. However, a 100 % reliability of the autonomous capabilities is practically unrealistic and the requirements in terms of safety are enormous since the robots are operated in direct vicinity of humans. Therefore, telepresence technologies are applied to cover several aspects (see Figure 11). For example, in case of emergency a teleoperator can instantly gain control of the remote robot and take immediate actions before an ambulance arrives on site. Alternatively, the person in need of care can activate teleoperated human assistance, if desired, or the robot requests human support itself if the autonomous capabilities of the system do not suffice to solve a required task.

The *SMiLE* ecosystem foresees haptic input devices to control a large variety of heterogeneous robotic agents in order to increase their reliability and efficiency. Therefore, a uniform control structure has been developed in which the robotic agents act at the users' requests, while seamlessly switching to remote haptic teleoperation can be performed at any time. Besides the teleoperation coupling, the methods of supervised and shared autonomy are also designed in a robot agnostic way. Within the *SMiLE* ecosystem, these operation modes are fused with the delayed teleoperation control structure to augment the human operator with the model-based capabilities of the robot-side intelligence. Similar to autonomous functions, advanced control methods such as hierarchical whole-body control (Dietrich and Ott, 2020), which are parameterized with the knowledge of the remote model, can help to increase the usability of the robotic system.

It was shown that seamless switching to teleoperation can be achieved through the application of one common Cartesian controller for teleoperation and autonomous operation modes on the remote robot side. Furthermore, in order to sequentially couple one haptic device to a variety of robotic systems and to augment the human operator with the shared-autonomy functionalities, the coupling has to be designed in the Cartesian space as well. The results of *SMiLE* further confirm that the shared-autonomy functionalities can be stably combined with the time-delayed telemanipulation framework if the generation of the respective fictitious force feedback is designed with passive characteristics, as was outlined in section 2.4.

A challenge apparent in domestic use cases is the large variety of different objects and tasks the system has to handle. Here, the human teleoperator can not only serve as a fallback solution for tasks unknown to the system but the data generated in these task executions can help to increase the functionality of the autonomous agent. To this end, we investigate task representations that enable the definition of new tasks through learning by demonstration approaches.

### 4.3. Telesurgery (Bilateral Control Concepts and Haptic Augmentation)

The demographic change and the accompanied continuous development of medical technology to enable high quality of life is an important driving factor for surgical robotics technology. Goals of robotically assisted surgical systems (RASS) are manifold, ranging within the enhancement of surgical treatments in terms of safety for patients and clinicians, patient outcome, and short convalescence. Already in the 1970s first concepts of RASS were considered based on telemanipulation (Alexander, 1973). Nowadays more than 7 million procedures have been performed assisted by RASS, many of them by the *da Vinci Surgical System (Intuitive Surgical Inc.)*, which embodies a telemanipulation system, similar to the envisioned system of the 1970s (Klodmann et al., 2020).

**Figure 12:**
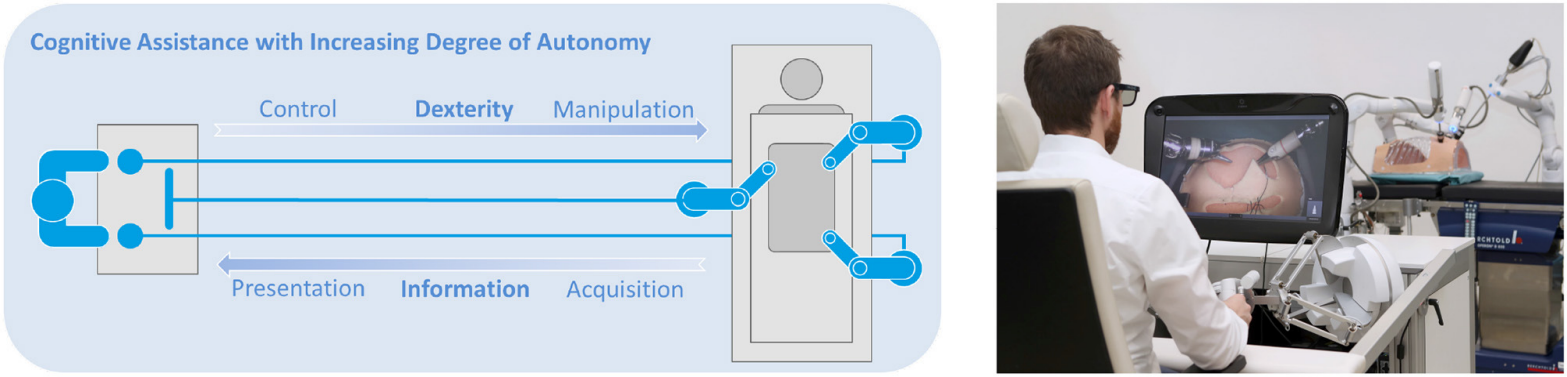
**(Left)** Focus areas of surgical robotics research. (translated by permission from Springer Nature Customer Service Centre GmbH: Springer Nature, (Klodmann et al., 2020), ©2020). **(Right)** DLR *MiroSurge* research system for telemanipulation in robot-assisted laparoscopic surgery. (©Alexandra Beier/DLR, CC BY 3.0).

Since the 1990s DLR contributes to this field, e.g., one of the most mature research platforms for telemanipulation in robotic surgery, the *DLR MiroSurge System*, was developed (Figure 12) (Hagn et al., 2010; Seibold et al., 2018). The modular patient-side manipulator consists of one to multiple bed-mounted 7-DoF *DLR MIRO* robot arms. One arm is equipped with a stereo endoscope and the others carry various types of articulated, wristed instruments (*DLR MICA*). The surgeon console incorporates a stereo display to visualize the situs of the patient in 3-D and two *sigma.7 (force dimension)* haptic devices are used as input devices (Tobergte et al., 2011). The system is the institute's core platform to research surgical robotics in interdisciplinary collaborations with industry, clinics, and complementary research institutions (MIRO Innovation Lab, 2017). Besides a seamless integration of RASS into a digitalized hospital infrastructure, the focus areas of research range from the acquisition and natural presentation of information from the situs to the surgeon, over enhancing the surgeon's dexterity inside the patient, while keeping the trauma low and providing natural controls, which assist with individualized and task-dependent assistance functions, e.g., utilizing virtual fixtures, shared control, or semi-autonomous functions, to further decrease the physician's cognitive workload (Figure 12 and sections 2.2 and 2.3).

The basic control architecture maps the user inputs to joint positions of the patient-side manipulators by an inverse kinematics algorithm accounting for workspace constraints, singularities, and redundancy. This basic control architecture is continuously enhanced by different MATM-based approaches, as described in the following paragraphs.

Different passivity-based force feedback control approaches to increase the system's transparency, e.g., by downscaling the felt inertia and friction and dealing with other disturbances, were developed (Tobergte et al., 2011; Tobergte and Albu-Schäffer, 2012; Tobergte and Helmer, 2013). Even though many studies show that force/torque feedback might increase also surgical performance (Weber and Schneider, 2014), cost effective, sterilizable sensors integrable directly at the instrument tips are still not commercially available.

Haptically augmented workspace limits (e.g., of the haptic device, the remote manipulators or task-dependent constraints, such as the incision point constraint), limit-indexing or velocity scaling approaches support the safe and efficient control of the system. To appropriately configure and parameterize these features, user-studies based on best practices and standards of human factors are conducted and are generally recommendable (Nitsch et al., 2012; Weber et al., 2013).

A rich set of geometric primitives is implemented to provide task-related and haptically augmented virtual fixtures that are intended to finally enhance the surgeon's capabilities, e.g., by guiding toward or along target tissues or preventing unintentionally injuring critical anatomical structures. Perceiving the patient's situs accurately and reactively update the robot's knowledge or rather representation of patient and procedure (section 2.4) to appropriately configure and parameterize these features embody some still open research questions to finally integrate the concept of task-dependent assistance functions into realistic scenarios in robot-assisted laparoscopic surgery.

### 4.4. Telenavigation (Haptic Augmentation and Model-Mediated Telemanipulation)

The ambitious future of planetary exploration has the potential to push the boundaries of technological advancements. However, nondeterministic remote environment at communication delay might render full autonomy and supervised control as an unfeasible feat with laborious task execution times. Instead adding human to the loop, to telenavigate the robot, can bypass many of the task requirements especially in the fields of perception and cognition, and ensure safety. Unlike telemanipulation, telenavigation benefits from velocity as the command signal, instead of position. Nonetheless, it too presents us with the trade-off between performance and stability.

**Figure 13:**
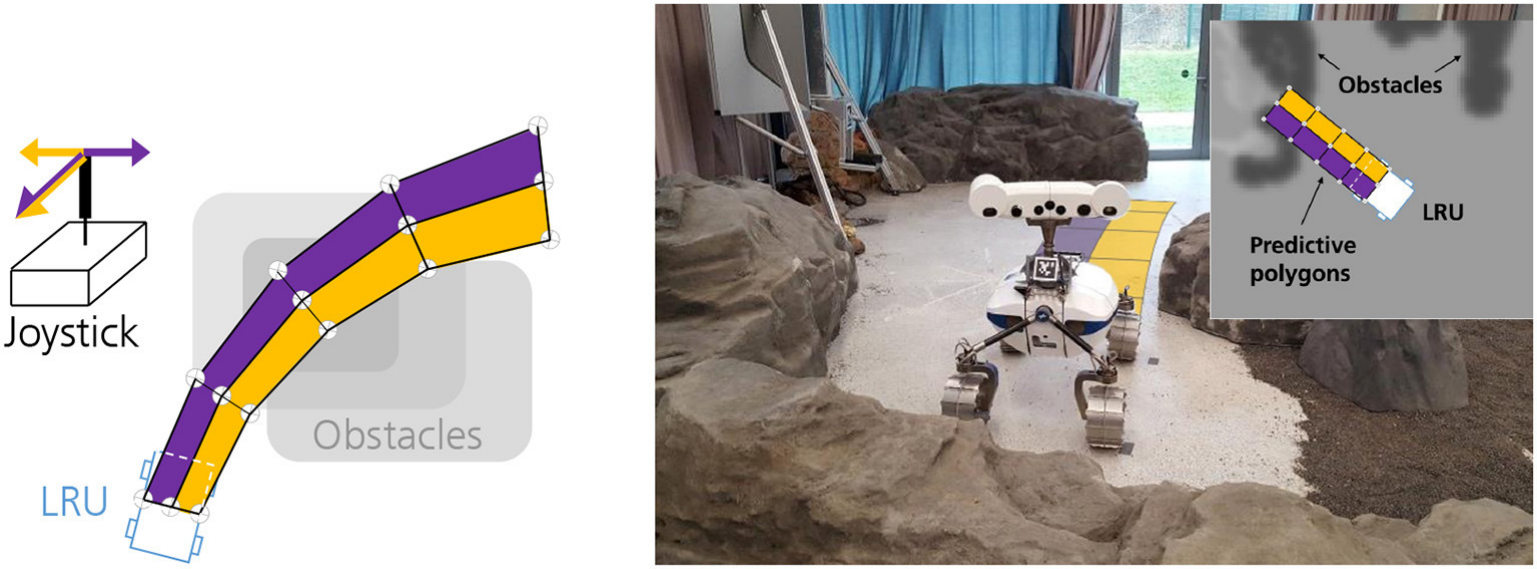
**(Left)** Schematic showing the generation of fictitious forces by polygons overlapping with obstacle in the danger map [©2020 IEEE. Reprinted, with permission, from Panzirsch et al. (2018b)]. **(Top Right)** Screenshot of the user interface. **(Bottom Right)** LRU with augmented polygons in the experimental environment [©Sierotowicz et al. (2020), CC BY 4.0, images have been modified].

To examine the effect of such a trade-off, a recent study (Sierotowicz et al., 2020) was conducted to telenavigate a Light Weight Rover (LRU; Wedler et al., 2015) with and without delay, via a 2-DoF DLR force feedback joystick (Riecke et al., 2016) by using a predictive polygon-based approach with a car-like interface (Panzirsch et al., 2018b) (Figure 13). TDPA was extended and used as a tool to passivate the active communication channel, which injects energy to the system due to delay. The human operator commanded longitudinal velocity and lateral curvature to the LRU, by pressing the dead-man switch on the joystick, and in return received a fictitious force feedback computed by the overlapping of polygons with the obstacles in a danger map. The danger map of the remote terrain is generated by classifying the traversability based on the depth data acquired by the LRU's pan/tilt stereo camera system (Brand et al., 2014), and is the local model that was used to generate haptic feedback (see section 2.2). A passive model update was achieved by Panzirsch et al. (2018b) (see section 2.4), which makes this a favorable approach in terms of applicability to a large variety of feedback generation types.

The main findings of the user study is that force feedback significantly improves navigation performance in the proximity of obstacles, although navigation is slower. The positive effect of the force feedback was evident in conditions without and with a communication delay of 800 ms. Altogether, these results show that a fictitious force feedback approach based on a TPDA controller is beneficial in difficult terrain and in the presence of substantial communication delay.

Apart from collision avoidance, the predictive polygon method could also help maintain a certain “safe” distance for the LRU from its environment. Since the width of the predictive polygons is a tunable factor, it can be adjusted to increase or decrease the safety factor or to allow/restrict the LRU's movement through narrow canyon-like environment. Despite of rate control, the TDPA could effectively stabilize and provide valuable force feedback with minimized position drift to the human operator. Thus, the haptic augmentation was beneficial with regard to navigation accuracy for demanding telenavigation tasks.

The 2-D danger map considers any object above a certain height as an obstacle. Thus, this would be impossible to tune when the LRU is traversing an unstructured environment. Therefore, a 2.5-D danger map with annotations would give more freedom to the operator and allow driving over small pebbles, grass, uneven roads, etc. Although a 2-DoF joystick could be used to maneuver the LRU with a car like interface, a 3-DoF haptic joystick could be used to fully explore LRU's potential of rear steering capabilities for crab-like and sideway motions.

### 4.5. Aerial Manipulation (Hierarchical Bilateral Teleoperation and Haptic Augmentation)

The use of aerial manipulators, i.e., unmanned aerial vehicles (UAVs) with attached robotic arms, allows for significant improvements in the reachability and versatility of manipulation tasks. Among other functionalities, such systems are able to perform inspection and maintenance tasks in high or inaccessible scenarios (e.g., oil refineries; Ollero et al., 2018). In order to exploit such systems while taking advantage of human capabilities in terms of perception and cognition, bilateral aerial teleoperation arises as a reasonable solution. In that scope, providing the user with camera images and/or virtual reality has been shown an essential feature for the successful fulfillment of the teleoperation task (Coelho et al., 2020; Lee et al., 2020).

**Figure 14:**
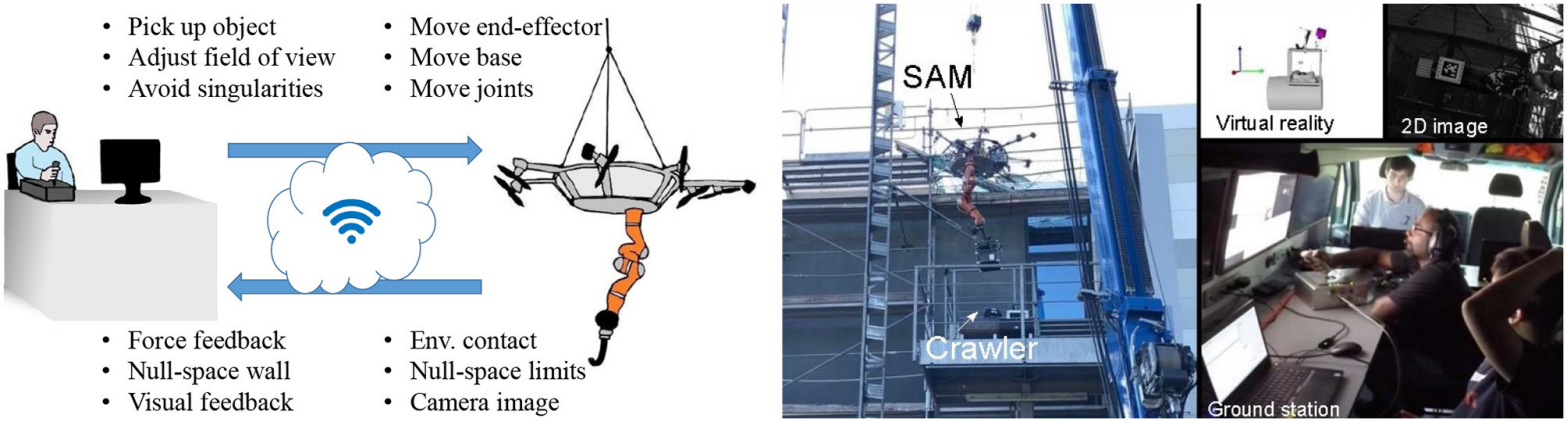
**(Left)** Concept of the whole-body teleoperation approach for the SAM. [©Coelho et al. (2021), CC BY 4.0]. **(Right)** Experimental setup, showing the robot, the ground station and the view provided to the user [©2020 IEEE. Reprinted, with permission, from Lee et al. (2020)].

Within the class of aerial manipulators, those presenting kinematic redundancy like the DLR Suspended Aerial Manipulator (SAM; Sarkisov et al., 2019; see Figure 14) are able to allow the user to not only control the robotic arm, but also steer the UAV (also called flying base) to achieve a desired camera view of the task being performed. Nevertheless, two main issues arise in that application. First, suitable control strategies have to be applied in order to ensure a strict hierarchy between the manipulation and the vision task, i.e., such that the flying base can move without disturbing the manipulation task. Additionally, as the traditional TDPA method is not capable of dealing with such a hierarchy in the presence of time delays, an extension thereof has to be applied.

An initial approach to cope with the aforementioned issues was introduced in Coelho et al. (2020) and a complete solution was subsequently presented in Coelho et al. (2021). A conceptual view of the presented approach is shown in Figure 14 together with an overview of an experimental scenario. Using the proposed approach, the user was able to choose to command either vision or manipulation task while the other task was autonomously controlled to keep the last commanded pose in a shared-control fashion. As the vision task was restricted to the motion subset where the end-effector is not disturbed, a haptic concept called *Null-Space Wall* was created to inform the user when the limits of that subset were reached. Moreover, the extended TDPA ensured the system passivity in simulations with up to 300 ms round-trip delay as well as in a real scenario, where command and feedback signals were exchanged through a wireless network with time delay, package loss, and jitter. The user was able to successfully perform pick-and-place tasks while keeping the manipulator and the object in the field of view. In addition, it was found that moving the flying base to align the camera image with the command directions of the input device can significantly decrease the task-completion time as well as the mental effort.

A current limitation of the proposed approach is that it does not take into account the constraints imposed by the cable system on the SAM. Therefore, it is only guaranteed to work when the oscillations of the base are negligible. An extension of the approach to deal with such constraints is planned for the near future. In addition, the visual-inertial odometry-based approach presented in Lee et al. (2020) to create a 3-D virtual-reality environment will be extended with haptic rendering capabilities. Moreover, the multilateral haptic augmentation method based on virtual grasping points (see section 2.2) could be especially meaningful in the described setup for the cases when the flying robot needs to keep some distance from obstacles. In that case, the robot grasping point on the manipulated object can be distant from the environment interaction point of the object, which can be chosen as the virtual grasping point.

### 4.6. On-Orbit Servicing (Shared Control)

**Figure 15:**
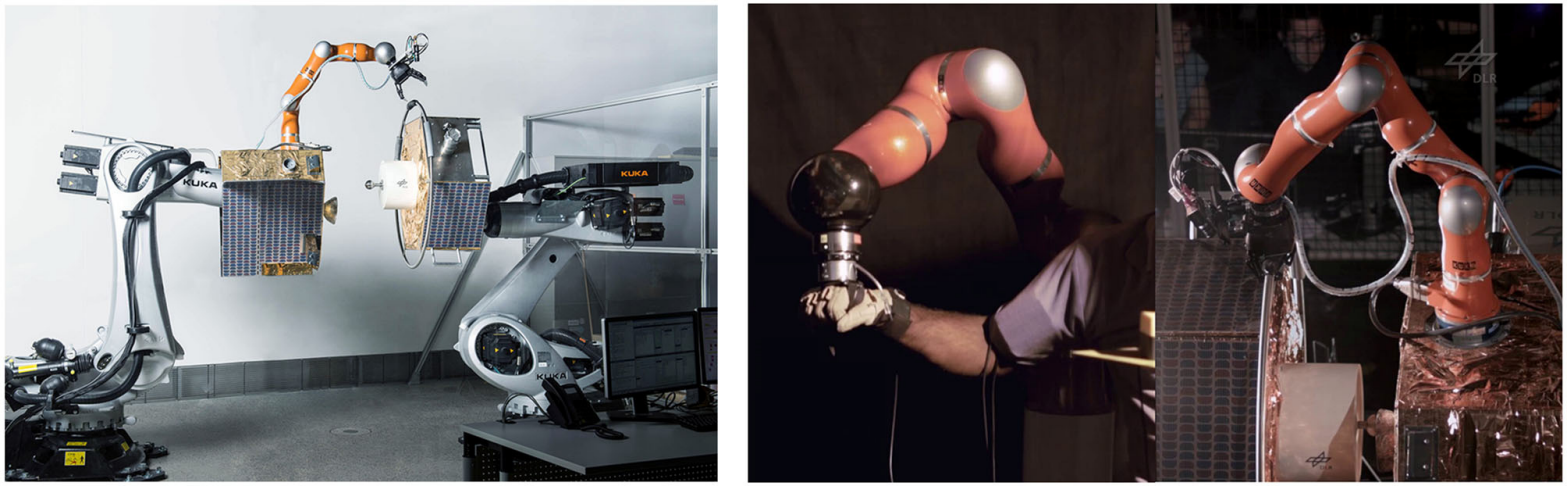
**(Left)** The On-Orbit Servicing facility at DLR. **(Right)** The haptic device and the remote robot are of the same type of collaborative robot.

Mitigation of space debris and servicing of dysfunctional satellites have driven space agencies and companies toward the concept of robotic on-orbit servicing (Miller et al., 1982). The term On-Orbit Servicing (OOS) refers to the maintenance in orbit, including assembly, refueling, and repair of defective satellites to extend their lifetime and to actively remove the space debris with a controlled re-entry into the Earth's atmosphere. To this end, space robotic projects consider the employment of a manipulator arm attached to a new satellite to implement the multiple phases of an OOS mission, namely, approaching a target satellite, followed by grasping, stabilization, docking, and finally servicing. In order to test and validate the low-level and high-level control strategies (aimed to be used in micro-gravity conditions) prior to the real mission, an on-ground facility to simulated the free-floating nature of the satellites. To achieve this, the OOS Simulator (OOS-Sim) hardware facility (Figure 15) has been developed at DLR, which comprises of two large industrial robots that simulate micro-gravity environment using model-based dynamic simulation for the satellite mock-ups attached to their end-effectors. The servicer satellite is equipped with a robot arm to service the target satellite mock-up (Artigas et al., 2015).

The manipulator arm attached to the satellite mock-up can be controlled using vision-based semi-autonomous control with the stereo camera set-up at the end-effector of the robotic arm. This semi-autonomous approach relies highly on perception of the target satellite, which is affected by internal and external factors such as camera noise, close range vision degradation, illumination changes, and reflections among many other (Schmidt et al., 2016). These factors might lead to a failure in the task execution by the autonomous system, and a human in the loop supervision is always preferred due to the critical nature of the orbital robotic missions.

To enable human intervention in the event of autonomy failure, the OOS-Sim also features teleoperation modality using a haptic device with which an operator can control the manipulator on the servicer satellite. To validate orbital teleoperation tasks in Low Earth Orbits (LEO) satellites with long operation windows using the OOS-Sim facility, experiments were conducted with the ASTRA GEO satellite acting as a relay system for the signals from the operator and the OOS-Sim manipulator, where the round-trip delay was 270 ms with standard deviation of 3 ms and a mean data loss of 24%. It was presented in Artigas et al. (2016a) that grasping and stabilization of the free-floating OOS-Sim target using the servicer manipulator with teleoperation is feasible even under large time delays and data losses.

## 5. Conclusion

Traditional bilateral teleoperation proved to be feasible up to time delays of several seconds round-trip (see section 2.1). However, task execution then becomes extremely difficult and slow. Particularly in scenarios with such long delays, a support of the operator by suitable technologies can be of great value. This paper introduced the MATM approach, which aims to enable efficient operator-assisting telemanipulation. The concept encompasses and generalizes previous approaches for enhanced telemanipulation, in particular model-mediated telemanipulation, shared autonomy, and augmented haptics. The approach employs two kinds of models to achieve this goal and to augment both the feedback to the operator and the commands for the teleoperated remote robot. In particular, a remote model enables a shared autonomous functionality of the teleoperated robot, while a local model aims at generating an assistive augmented haptic feedback to the human operator. This scope makes the MATM approach one of the most comprehensive and powerful, but also one of the most technologically sophisticated and challenging telemanipulation approaches.

In a historical retrospective of our past telerobotic space missions, the way to this technology was described and the challenges we encountered during these missions were highlighted. The biggest challenge of the first missions we participated in, ROTEX and EST VII, was to overcome the hurdles that were imposed by the low computing power at that time that led to long time delays. Since these delays made closed-loop telemanipulation with force feedback impossible, our research concentrated on shared control and model prediction. Later, in ROKVISS and Kontur-2, the development of a control approach that allows stable and transparent bilateral telemanipulation despite delay, loss, and jitter of communication packets became the main focus of our research activities on telemanipulation. During the latter mission, basic research on the design of optimal haptic feedback was also conducted. In the more recent METERON mission, supervised autonomous operation was evaluated using a humanoid robot as an exemplary execution platform. It turned out during this mission that such an autonomous functionality can provide a great relieve for operating a robot and even allows for parallel operation of several robots. However, it also showed the limitation of autonomous operation especially in unstructured environments. Without human perception and cognition, a robot system will in the near future not be able to operate autonomously during a whole mission, although autonomy can already perform some specific robotic tasks today. These results suggest to combine autonomy and telemanipulation in an advantageous way, which is realized in particular with the remote model of MATM. In the recent Analog-1 mission, the telemanipulation technologies for the telenavigation of a rover through an unstructured environment and for the teleoperation of a robot arm mounted on this rover were evaluated. It could be shown for the first time that full-DoF direct teleoperation with force feedback can be robustly established for such a system and underlines the benefit of haptic feedback over open-loop teleoperation.

While these space missions were the main driver for our research on telemanipulation, the technology also has enormous potential for other applications, which was highlighted on the basis of six use cases. These use cases unveiled the potential and limitations of the MATM approach in the applications that reach from orbital applications over terrestrial telemanipulation in caregiving and telesurgery to applications that involve driving and flying robotic systems. In none of these use cases have we exploited the full spectrum of MATM so far, but rather emphasized certain aspects of it. The following lines give an overview of the most important results.

Haptic augmentation methods, in particular task- and system-related virtual fixtures demonstrated their usefulness in telesurgery, caregiving, and orbital robotics. In telenavigation, a predictive polygon method helped to maintain a certain “safe” distance for DLR's rover LRU from its environment and therefore to avoid collisions. We identified an enormous potential in making haptic augmentation methods more flexible, which could be achieved by parameterizing them manually using human intervention or automatically by machine learning methods.

While in the presented use case of aerial manipulation, the autonomous functionality took control of a subtask and was thus used to support the operator, the shared control for on-orbit servicing showed that even proactive autonomous trajectory generation is comprehensible and clearly supportive for the operator. The mixed-initiative-based shared control present certain limitations, particularly if object recognition is affected by camera noise, close range vision degradation, illumination changes, and reflections. More adaptive approaches need to be developed in future enabling easier human intervention or automatic adaption of authority. With regard to the caregiving use case, it is apparent that the shared control approach is currently only able to handle objects that are previously known to the algorithm. To overcome this limitation, in the future new objects could be self-learned by learning-by-demonstration approaches.

With regard to stability, we could confirm in the caregiving use case that the combination of shared-autonomy functionalities and a time-delayed telemanipulation framework becomes stable if the respective fictitious force feedback is designed with passive features. All our available MATM modules for delayed teleoperation, bilateral, or multilateral haptic augmentation methods and model updates were implemented on the basis of passive modules, which can be almost arbitrarily combined without further stability considerations. Furthermore, seamless switching from autonomy to telemanipulation or between different teleoperated robots was enabled by a coupling control structure that can be readily transferred to comparable telerobotic systems as well.

Beyond these lessons learned and the challenges identified, a number of other important questions remain for future work, in particular to enable the MATM approach to be realized as a whole, incorporating all of its technologies. A robust and powerful solution for updating the symbolic models for supervised autonomy especially during the teleoperation phase is still an active topic of research. In relation to this task, the synchronization between the local and the remote model also needs to be developed. The passivity principle, which we applied to achieve stability, needs to be validated as a suitable tool for a general control framework to allow easy extension of the MATM approach for new applications and robots. Furthermore, a new transparency metric would be useful for comparing MATM with direct teleoperation methods and model-mediated teleoperation. Finally, as a big challenge remains the design of a user interface involving graphical, audio, and haptic channels that provides the operator access to all model-augmentation functionalities and control modalities reaching from direct teleoperation to supervised autonomy. While MATM has not been implemented as a whole, it has already proven its usefulness in numerous applications and plays an important role as intermediate step toward supervised and fully autonomous robots.

## Data Availability Statement

The original contributions presented in the study are included in the article/supplementary material, further inquiries can be directed to the corresponding author/s.

## Ethics Statement

Written informed consent was obtained from the individual(s) for the publication of any potentially identifiable images or data included in this article.

## Author Contributions

TH: contributed to all sections. AA-S: wrote section 1. GH: wrote section 3.1. CO: wrote section 2.1. DL: wrote sections 2.3 and 2.4. AD, JV, KH, AH, and JK: wrote section 4.3. RL: wrote section 4.6. MS: wrote section 4.4. AB: wrote sections 1, 2.3, and 2.4. GQ: wrote sections 2.2, 2.3, and 2.4. NL: wrote section 3.3. BB: wrote sections 3.1 and 3.2. CR: wrote section 3.2. NB: wrote section 2.1. BW: wrote sections 3.2, 4.3, and 4.4. AP: wrote section 3.4. AC: wrote section 4.5. RB: wrote sections 3.2, 4.1, 4.4, and 4.6. HS: wrote sections 1, 2.1, 4.4, and 5. MP: wrote sections 2.1 and 5. All authors contributed to the article and approved the submitted version.

## Conflict of Interest

The authors declare that the research was conducted in the absence of any commercial or financial relationships that could be construed as a potential conflict of interest.

## Footnotes

^1^The acronym SMiLE stands for service robotics for people in restricted living situations (in German “Servicerobotik für Menschen in Lebenssituationen mit Einschränkungen”).
